# Minimal Incision Repair of Rectus Abdominis Diastasis (MIRRAD) as day-case surgery: A prospective study

**DOI:** 10.1007/s10029-025-03306-x

**Published:** 2025-04-15

**Authors:** Asmatullah Katawazai, Göran Wallin, Gabriel Sandblom

**Affiliations:** 1 Department of Surgery, Faculty of Medicine and Health, University Hospital Örebro, Karlskoga Lasarett, School of Medical Sciences, Örebro University, 691 44 Karlskoga, Sweden; 2 Department of Surgery, Faculty of Medicine and Health, University Hospital Örebro, School of Medical Sciences, Örebro University, Karlskoga, Sweden; 3 Department of Clinical Science and Education Södersjukhuset, Department of Surgery, Södersjukhuset, Karolinska Institute, Stockholm, Sweden

**Keywords:** Rectus diastasis, Ventral hernia, Linea alba, Postpartum rectus diastasis, Minimal incision surgery

## Abstract

**Purpose:**

Postpartum rectus abdominis diastasis (PP-RAD) is a condition that may cause abdominal wall insufficiency, affecting daily life. When conservative treatments are unsuccessful, surgical intervention may be necessary. This study aimed to assess the safety and effectiveness of minimal incision repair of rectus abdominis diastasis (MIRRAD) as day-case surgery in women with PP-RAD.

**Methods:**

This study included 33 female patients aged 20–50 years with PP-RAD and an inter-rectus distance (IRD) of ≥ 3 cm. All patients had previously undergone conservative treatment without satisfactory outcomes. Each patient received the MIRRAD procedure as day-case surgery apart from one who stayed overnight due to nausea. Follow-up evaluations were conducted at 4 h, 1 week, 1 month, and 1 year after surgery.

**Results:**

The average inter-rectus distance (IRD) was 4.4 cm, with a mean diastasis length of 15 cm. Of the 33 patients included, 2 did not attend the 1 year follow-up leaving 31 for final analysis. Of these, 30 had one or more concomitant hernias. The mean operation time was 67 min. At the 1 year follow-up, 87% of patients were satisfied with the results, and 90% said they would undergo the procedure again if necessary. No surgical site infection was reported, and recovery was generally smooth. Thirty of the 31 patients were discharged within 4 to 6 h after surgery, while one patient stayed overnight.

**Conclusion:**

MIRRAD appears to be a safe and effective surgical option for PP-RAD, particularly in cases without significant excess skin. Further studies with larger populations and longer follow-up are needed to confirm these findings and establish standard patient selection criteria.

**Supplementary Information:**

The online version contains supplementary material available at 10.1007/s10029-025-03306-x.

## Introduction

Rectus Abdominis Diastasis (RAD) refers to separation of the rectus abdominis muscles by more than 2 cm, leading to a widening of the linea alba—the connective tissue that runs along the midline of the abdomen[1–3]. This condition is often triggered by factors that increase intra-abdominal pressure and by hormonal changes, particularly during pregnancy. As the uterus expands, it can stretch the abdominal wall, resulting in RAD. In some cases, this separation persists after childbirth, known as postpartum rectus abdominis diastasis (PP-RAD). PP-RAD can lead to significant functional challenges due to instability and weakness of the abdominal wall, which compromises the structural integrity of muscles and fascia. This instability can diminish core strength and trunk function and may also impact the pelvic floor muscles [4, 5]. Symptoms of PP-RAD are both direct and indirect. Direct symptoms include a noticeable bulge along the midline of the abdomen, especially visible during movements that involve the abdominal muscles such as lifting or sit-ups, causing discomfort and a sense of weakness. Indirect symptoms such as lower back pain, pelvic pain, and urinary incontinence are due to decreased support from the abdominal wall and pelvic floor muscles, which are essential for maintaining proper posture and stability. Research indicates that these symptoms can substantially interfere with daily tasks, physical activity, and overall physical function, lowering the patient’s quality of life [6]. Furthermore, PP-RAD increases the likelihood of developing a hernia along the linea alba and can contribute to postural problems such as hyperlordosis [4, 7]. A recently published literature review including 37 studies conclude that both open and laparoscopic approach to repair RAD are safe. The study also found that a double-layer suture technique was associated with lower rate of complication [8].

The most widely used surgical method for PP-RAD involves a significant procedure with a large incision extending from one spina iliaca anterior superior (ASIS) to the other, often combined with abdominoplasty to remove excess skin. This type of surgery typically may require a hospital stay of one or several days and carries the risk of both intraoperative and postoperative complications. A meta-analysis of 4,295 abdominoplasty cases described an overall complication rate ranging from 9.3% to 33.8%, with revision rates between 3 and 22% [9]. Given these risks, there is a clear need for a less invasive surgical alternative. This study aimed to assess the safety and efficacy of Minimal Incision Repair of Rectus Abdominis Diastasis (MIRRAD) as a day-case surgical option in selected patients. The study included 33 women with PP-RAD ≥ 3 cm, focusing on primary outcomes such as improvements in physical health and overall life satisfaction. Two patients were lost to the study leaving 31 for analysis. The findings suggest that a minimal incision technique can provide a safer and less invasive alternative to conventional surgical methods, with shorter recovery time and lower complication rate.

## Method

### Study design and materials

In this prospective study, 33 female patients diagnosed with postpartum rectus abdominis diastasis (PP-RAD) with an inter-rectus distance of ≥ 3 cm were initially enrolled. All participants had previously undergone conservative treatments without satisfactory results and chose to undergo surgical repair using the MIRRAD procedure. Prior to participation, each patient was provided with detailed verbal and written information about the study, and gave their written consent to participate. The rectus diastasis was measured 1 cm above the umbilicus in all participants. All patients underwent a clinical examination, and if any doubts arose, they were referred for a CT scan. Patients were instructed to perform a slight crunch position to allow the examiner to palpate the edges of the rectus muscles on both sides. While lying on their back with straight legs, the examiner marked the edges of the rectus muscles along the linea alba using a marker pen. The inter-rectus distance was then measured with a measuring tape. None of the study participants had a history of previous surgery involving the linea alba.

The same procedure was followed for patients who underwent a CT scan, ensuring consistency in measurements. Additionally, the measurements were repeated during surgery to confirm the inter-rectus distance.

Two patients did not attend the 1 year follow-up: one due to long distance and time constraints, and the other without providing a reason. Both patients declared no complications at earlier postoperative follow-ups. Thirty-one patients attended the 1 year follow-up.

Three women had asthma, two had hypertension, one had diabetes, and one woman had mild multiple sclerosis.

The number of pregnancies, ranged from 1 to 8 pregnancies with the mean 2.9 (SD 1.7). The patients, aged 20 to 50 years, had a mean body mass index (BMI) of 24.1 (range: 18.8–30.1), with a standard deviation of 2.8. Their occupations varied, with 68% involved in physically demanding or light labour and 32% working in an office setting. Surgery was performed as a day-case procedure, with all but one patient being discharged within 4–6 h after surgery. One patient stayed overnight due to nausea, but no postoperative complication or readmission was reported.

Data were collected electronically using Greenlight Guru Clinical software (formerly Smart-Trial) and exported to Excel for further analysis in IBM SPSS Statistics version 29.0. Participants' current illnesses, classified using ICD codes, and ongoing medications were recorded. All patients were examined by the operating surgeon 4 h postoperatively. Subsequently, all patients were scheduled for a video follow-up with an experienced nurse using the Visiba Care software application. Each patient was physically examined by an experienced surgeon before surgery and at follow-up visits 1 month and 1 year postoperatively. A surgeon was consulted in the case of any surgical site issues.

**Table 1:** Baseline data.

	N	Minimum	Maximum	Mean	Std. Deviation
Hernia size cm	30	0.4	1.2	0.7	0.21
Suture-line length in cm	31	12.0	20.0	15.1	2.0
Peroprative Inter-rectus distance in cm	31	3.0	8.0	4.4	0.9964
Peroperative length of diastasis in cm	31	12	20	15.9	2.6
Operation time in minutes (skin to skin)	31	35	120	67.3	19.7
Valid N	31

**Table 2:** Case Summaries, per-operative measurements of all patients.

ID		Length of diastasis in cm	Inter-rectus distance in cm	Suture-line length in cm after repair
1		13	4.5	13.0
2		19	5.5	18.0
3		17	4.5	17.0
5		15	3.5	14.0
6		12	3.0	13.0
7		20	5.5	20.0
8		14	4.0	14.0
9		14	4.0	12.0
10		20	3.5	17.0
11		12	3.4	12.0
12		14	4.5	14.0
13		20	8.0	15.0
14		13	4.5	14.0
16		16	5.0	14.0
17		18	4.0	16.0
20		17	5.0	17.0
21		12	4.0	14.0
22		15	5.0	16.0
23		15	4.0	13.0
25		12	4.0	12.0
26		16	3.2	16.0
27		18	5.0	18.0
28		16	4.7	14.0
29		13	3.5	13.0
30		17	4.5	16.5
31		17	4.0	15.0
32		19	6.0	16.0
33		17	4.5	17.0
34		15	3.0	15.0
36		20	5.0	17.5
37		18	4.5	17.0
Total	N	31	31	31

### MIRRAD-procedure step-by-step

**Fig. 1:**
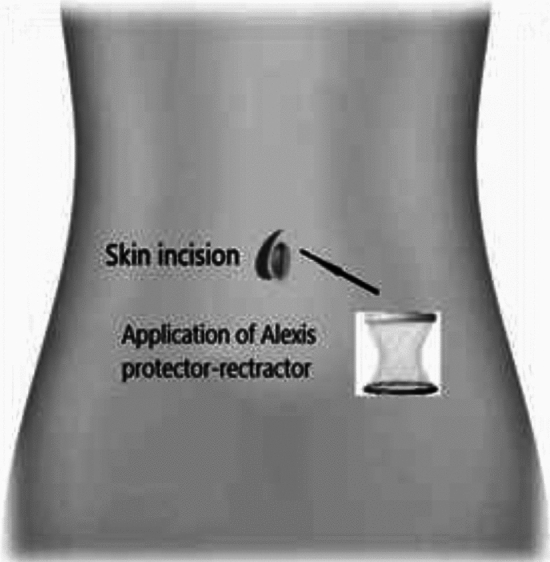
Skin incision and application a retractor

**Fig. 2:**
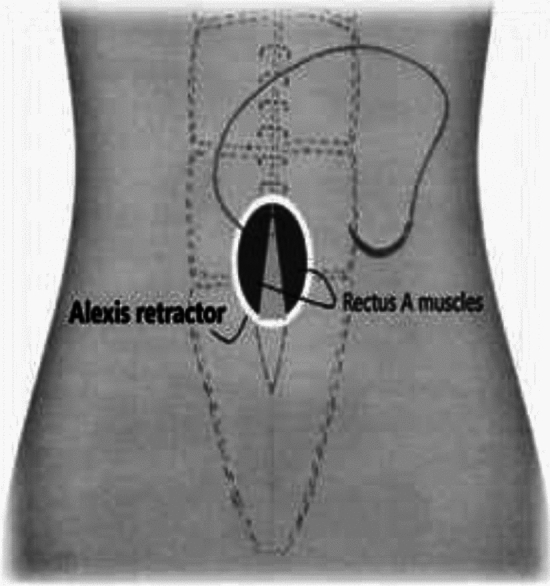
Plication of rectus diastasis

**Fig. 3:**
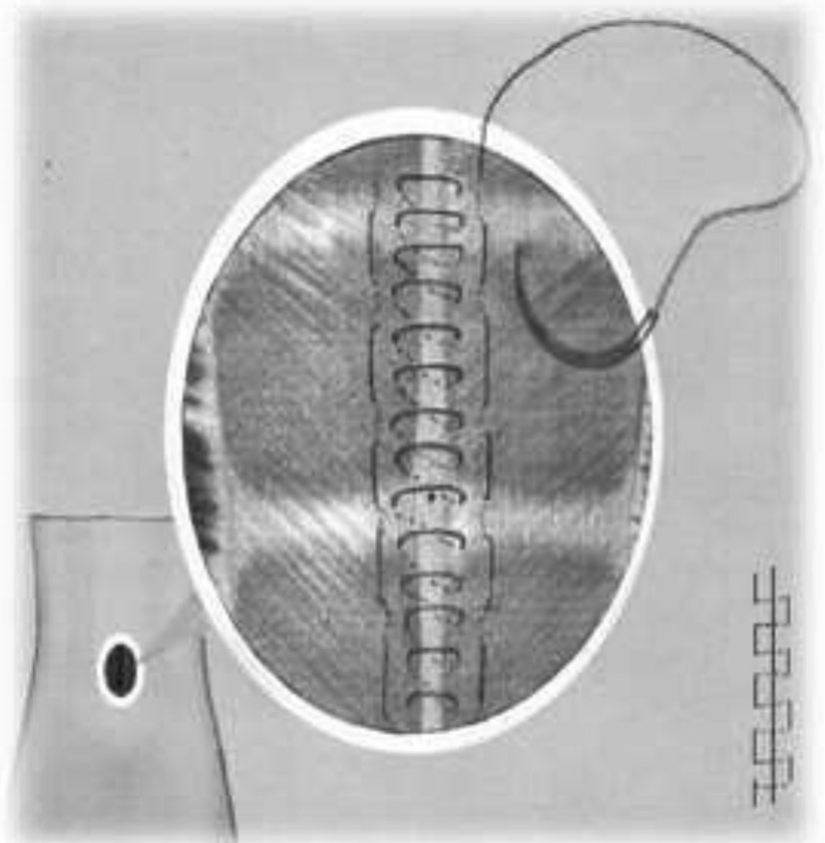
Reinforcement with a second suture line

**Fig. 4:**
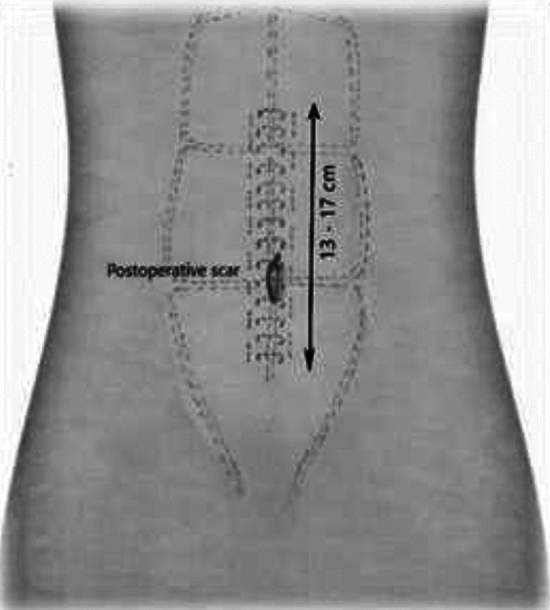
postoperative scar and Final result

**Fig. 5:**
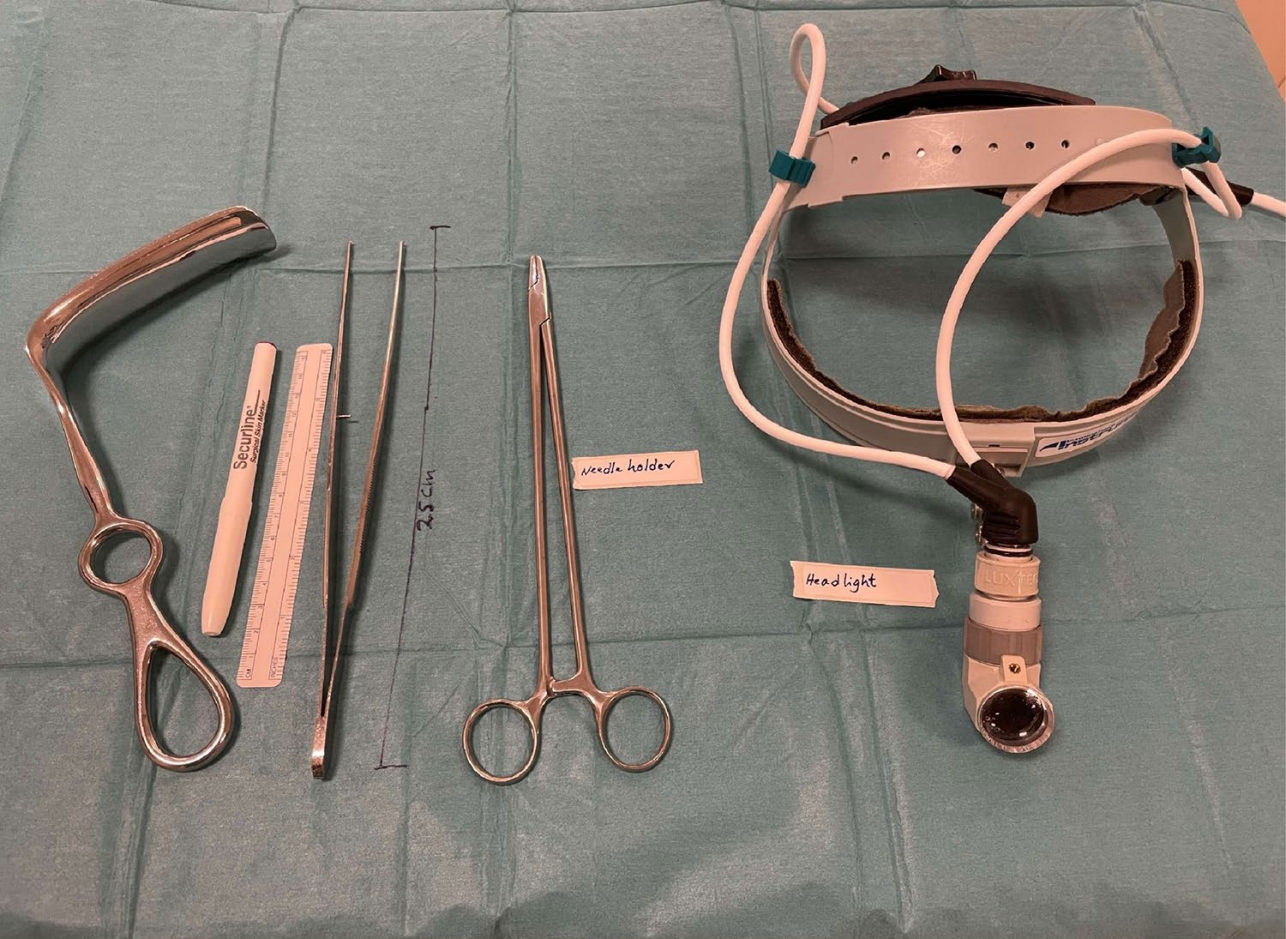
Surgical instruments used in the MIRRAD procedure. An extra-long needle holder and forceps, along with a headlight and Doyen retractor

Minimal Incision Repair of Rectus Abdominis Diastasis [MIRRAD] is a novel surgical technique performed as day-case surgery. The procedure is performed under general anaesthesia. A 3 cm skin incision is made on the right side of the umbilicus extending to about 1 cm above the umbilicus. The umbilicus is then detached from the fascia. A circular plastic protector-retractor is applied. Subcutaneous tissue is free-dissected from the fascia along the linea alba approximately 8–10 cm above and 5–7 cm below the umbilicus, until the rectus muscle diastasis is completely exposed. The diastasis is then marked with a sterile marker and measured. Any concomitant hernia in the linea alba is repaired with a 2–0 prolene suture before repairing the rectus diastasis. An extra-long needle holder and forceps are required for the procedure. A continuous 0-PDS barbed suture technique is used to bring the separated rectus muscles together and reinforced with a second line of 0-PDS barbed suture as shown in Figs. 1, 2, 3, 4, 5. Local anesthetic [Ropivacaine 7.5 mg/ml] is injected alongside the suture line and skin incision. The umbilicus is then re-attached to the facia with single stitch 4–0 PDS and the skin sutured with intradermal resorbable 3–0 Monocryl. An abdominal binder is use postoperatively to prevent hematoma and seroma formation. Our approach did not aim to extend the repair to the xiphoid, as described in other studies. Instead, we focused on repairing the pathological portion of the RD (inter-rectus distance > 2 cm). The MIRRAD procedure was performed exclusively using an open surgical approach, without the use of laparoscopic instruments. The surgeon utilized a headlight for better visibility during the procedure. Basic surgical instruments were employed, with extra-long tools such as needle holders and forceps being used to facilitate the repair. A video of MIRRAD surgical technique was submitted and presented in European Hernia Society (EHS) annual congress, at Barcelona 2023.

### Statistical analyses

The Greenlight Guru Clinical (formerly Smart-Trial) was used to register all data electronically. Upon completion of the study, the data were exported to IBM SPSS version 29.0 for analysis. Wilcoxon Signed Rank test was used for comparison of pre- and postoperative data. Descriptive statistics and correlation analyses were performed for a variety of different data analyses.

Three women had asthma, two had hypertension, one had diabetes, and one woman had mild multiple sclerosis.

The correlation between the number of pregnancies and the rectus abdominis diastasis (RAD) width at the operation was 0.27, based on 31 observations, with a 95% confidence interval from − 0.09 to 0.57. Similarly, the correlation between the number of pregnancies and RAD length was 0.16, with a 95% confidence interval from − 0.20 to 0.49. Despite the wide variation in number of pregnancies, ranging from 1 to 8 pregnancies, no significant correlation was found between the number of pregnancies and the size of the RAD.

The mean time for the surgical procedure was 67 min (SD 19.7 min). The mean width of the diastasis was 4.4 cm (SD 1.0 cm), and the mean cranio-caudal length of the diastasis was 15 cm (SD 2.6 cm). All patients had at least one concomitant hernia, and three patients had two or more hernial defects. The mean length of the linea alba suture line was 15.2 cm (SD 2.0 cm) Tables 1, 2.

### Follow-up

All study patients were followed 4 h postoperatively, as well as at 1 week, 1 month, and 1 year after the procedure. If there was any clinical sign of a complication or a surgical site event, this was investigated at an extra visit, by laboratory tests, or by radiology. All events related to the procedure were registered in Greenlight Guru Clinical [formerly Smart-Trial], and the patients attended extra visits as per local routine. All patients were provided with an abdominal binder and advised to use it for 6 weeks postoperatively. The recovery period was consistent with expectations, with all patients experiencing mild to moderate postoperative pain and temporary limitations in physical activity. No severe complication was observed.

## Results

Of the 33 patients who underwent the MIRRAD procedure, 31 attended the one-year postoperative follow-up. The mean BMI was 24.1 (range 18.8–30.1) with a standard deviation of 2.8. Among the women included, 68% had physically light or heavy work, while 32% worked in an office setting. The procedure was performed as day-case surgery, allowing all patients to be discharged within 4–6 h after surgery, except for one patient who stayed overnight due to persistent nausea. There was no readmission due to a postoperative event, and no complication was reported. All patients were followed up by a nurse at 4 h, 1 week, and 1 month postoperatively. A surgeon was consulted in the event of a surgical site issue.

Postoperative pain was measured using a Visual Analogue Scale (VAS), with mean pain scores of 2.8 at 4 h, 3.2 at one week, and 1.7 at 1 month after surgery. Pain levels ranged from 1 to 9 at 4 h, 1 to 7 at one week, and 1 to 7 at 1 month, with standard deviations of 2.1, 1.5, and 1.3, respectively. Postoperative nausea was reported by one patient (3.2%) at 4 h, five patients (16%) at 1 week, and two patients (6%) at 1 month. Two patients experienced seroma-related swelling [one at 1 week and the other at 1 month], confirmed by radiology. One patient was readmitted 1 week after surgery with radiologically verified diverticulitis and was treated with antibiotics. This patient also underwent seroma aspiration to exclude surgical site infection (cultures were negative). No cases of surgical site infection (SSI) was reported.

One year postoperatively, the Ventral Hernia Pain Questionnaire (VHPQ) was used to assess pain. All study participants had one or more concomitant hernias in the linea alba, which may have contributed to their preoperative pain. Any concomitant hernias were repaired during the same procedure. One year postoperatively, pain levels were significantly reduced compared with preoperative levels.

A Wilcoxon Signed Rank Test was conducted to compare preoperative pain levels with those reported one year after surgery. Results showed that 28 of 31 participants experienced less pain one year after the operation, with a mean rank of 15.4 and a sum of ranks of 431.5. One participant reported increased pain, with a mean rank of 3.5, while two participants reported no change in pain level. The test revealed a statistically significant reduction in pain one year after surgery, with a Z-value of −4.7 and a two-tailed significance level of < 0.001.

Patient satisfaction was high, with 87% (27 out of 31) expressing satisfaction with the results, while 13% (4 out of 31) were dissatisfied, primarily due to persistent skin bulging. When asked if they would undergo the operation again if necessary, 90% (28 out of 31) responded positively, while 10% (3 out of 31) said they would not.

At the one-year follow-up, four patients reported mild pain or discomfort at the operation site, but none required pain medication or took sick leave All procedures were financed by the public health insurance.

## Discussion

The present study evaluated the safety and effectiveness of minimal incision repair of rectus abdominis diastasis (MIRRAD). The results show the potential of this minimally invasive technique in addressing both ventral hernia and functional concerns associated with rectus diastasis.

### Safety and recovery

The MIRRAD procedure was generally well tolerated, with minimal postoperative complication risk. Most patients were discharged within 4–6 h. The procedure may thus be performed as a day-case surgical procedure. The rapid recovery and low complication rate—largely limited to seroma and nausea—underscore the procedure's safety. There were no cases of surgical site infection, which further supports the procedure's safety profile.

### Effectiveness

The effectiveness of MIRRAD in patients with RAD and a concomitant hernia is evident from the significant reduction in pain and discomfort reported by patients at the one-year follow-up (VHPQ). The Wilcoxon Signed Rank Test revealed a substantial decrease in pain level (p < 0.001), indicating that the procedure effectively addresses the primary symptoms of rectus diastasis. Furthermore, most patients experienced improved abdominal wall strength and stability, enhancing their ability to engage in physical activities.

### Patient satisfaction

At one-year follow-up, 87% of participants stated that they were satisfied with the surgical outcome and 90% were willing to undergo the procedure again if necessary. This high level of satisfaction is indicative of the procedure's positive impact on patient quality-of-life, body image, and self-confidence. The significant reduction in abdominal pain and improvement in function likely contributed to these positive perceptions.

### Comparison with traditional techniques

Compared to established open surgical techniques, MIRRAD offers several advantages. The minimal incision approach results in a shorter recovery time, reduced postoperative pain, and fewer complications. Traditional techniques, while effective, usually require a hospital stay and are associated with higher morbidity [10]. The findings of this study suggest that MIRRAD can achieve comparable, if not superior, outcomes with a less invasive approach.

### Limitations

While the results are encouraging, the study has several limitations. The sample size is relatively small, and the follow-up period is limited to one year. Larger studies with control groups and longer follow-up periods are needed to confirm these findings and assess the long-term durability of surgical outcomes. Furthermore, the study cohort consisted solely of postpartum women, which may limit the generalizability of the results to other populations. The MIRRAD method may not be aesthetically the best choice when a patient has excessive saggy skin.

### Future directions

Future research should focus on comparing MIRRAD with other minimally invasive techniques, such as laparoscopic repair, as well as traditional open surgical methods, to establish a clear consensus on the most effective and safe approach for rectus diastasis repair. Studies should also investigate the long-term effects of the procedure on abdominal wall function, hernia recurrence, and overall quality of life. The development of standardized criteria for patient selection could further optimize outcomes and reduce complication rates.

### MIRRAD in context of other techniques

In the context of minimally invasive techniques for rectus diastasis repair, the MIRRAD method aligns with the growing trend of reducing surgical trauma while maintaining effective outcomes. Various innovative techniques have been developed to address rectus diastasis and hernias, each offering unique benefits and limitations.

The EMILOS (Endoscopic Mini/Less Open Sublay Repair) technique combines the advantages of open and laparoscopic approaches by accessing the posterior rectus sheath through a small incision [11, 12]. Mesh placement in the sublay position helps reinforce the abdominal wall, providing reduced postoperative pain and lower recurrence rates compared to traditional open repairs. This technique may be a preferred method in larger hernia defects with higher recurrence risk. MIRRAD is a much more easily performed procedure with shorter learning curve and basic surgical instruments.

Similarly, the eTEP (Enhanced View Totally Extraperitoneal Repair) method focuses on ventral and incisional hernia repairs by creating access to the retromuscular space without entering the peritoneal cavity[13, 14]. This technique ensures minimal trauma to the skin, but not to the abdominal wall. This technique is more suitable for larger hernia defects, allowing for large mesh placement while preserving the integrity of surrounding structures. The eTEP approach is a time consuming and technically difficult method as compared to MIRRAD's emphasis on precision and preserving functionality, suitable in classical postpartum RD with a small hernia.

The SCOLA (Subcutaneous Onlay Laparoscopic Approach) shares a hybrid approach like MIRRAD, where a subcutaneous working space is created through a small incision. However, SCOLA typically incorporates mesh reinforcement in the onlay position, avoiding the need for intraperitoneal dissection [15, 16]. While MIRRAD does not involve mesh placement, the SCOLA method demonstrates a similar commitment to reducing patient recovery time and postoperative discomfort. The MIRRAD could be performed with basic surgical stings, without needing the luxury of laparoscopy.

The REPA (Robot-Assisted Endoscopic Preperitoneal Approach) technique makes use of robotic technology to enhance the precision and visualization of rectus diastasis repairs [17]. This method implies placing the mesh in the preperitoneal or retromuscular position, combining the benefits of minimally invasive surgery with advanced instrumentation. Although MIRRAD is an open approach, the goals of minimizing invasiveness and optimizing surgical outcomes align closely with REPA. Although REPA is a good approach in complex abdominal wall hernias, it’s a very expensive and technically difficult approach.

The ELAR (Endoscopic-Assisted Linea Alba Reconstruction) technique focuses specifically on linea alba reconstruction for diastasis recti and hernias. By combining endoscopic tools with suturing techniques to approximate the rectus muscles, ELAR addresses both functional and aesthetic concerns. Mesh reinforcement is optional depending on the severity of the condition. The simplicity of MIRRAD, avoiding advanced instrumentation or mesh use, offers an alternative for cases where such complexities may not be required.

## Conclusion

The MIRRAD procedure appears to be a safe and effective treatment for rectus abdominis diastasis, with or without concomitant ventral hernia, offering significant benefits in terms of pain reduction, recovery time, and patient satisfaction. The minimal incision of the procedure, combined with positive outcomes, suggests that MIRRAD could become the preferred surgical option for patients with rectus diastasis. However, further research is required to validate these findings and refine the technique for broader clinical application. Studies with larger sample sizes and longer follow-up periods should be carried out to validate these findings and refine patient selection criteria.

## Supplementary Information

Below is the link to the electronic supplementary material.

Supplementary file1 (MOV 431643 KB)

## Abbreviations

MIRRAD: Minimal incision repair of rectus abdominis diastasis
RAD: Rectus abdominis diastasis
MATFP: Modified abdominal trunk function protocol
VAS: Visual analogue scale
DRI: Disability rating index
UDI-6L: IIQ-7 Urogenital Distress Inventory-6, Incontinence Impact Questionnaire -7
VHPQ: Ventral hernia pain questionnaire
SSIL: Surgical site infection
